# Genetic Diversity and Ecological Niche Modelling of Wild Barley: Refugia, Large-Scale Post-LGM Range Expansion and Limited Mid-Future Climate Threats?

**DOI:** 10.1371/journal.pone.0086021

**Published:** 2014-02-05

**Authors:** Joanne Russell, Maarten van Zonneveld, Ian K. Dawson, Allan Booth, Robbie Waugh, Brian Steffenson

**Affiliations:** 1 Cell and Molecular Sciences, The James Hutton Institute, Invergowrie, Scotland, United Kingdom; 2 Regional Office for the Americas, Bioversity International, Cali, Colombia; 3 Department of Plant Pathology, University of Minnesota, Saint Paul, Minnesota, United States of America; China Agricultural University, China

## Abstract

Describing genetic diversity in wild barley (*Hordeum vulgare* ssp. *spontaneum*) in geographic and environmental space in the context of current, past and potential future climates is important for conservation and for breeding the domesticated crop (*Hordeum vulgare* ssp. *vulgare*). Spatial genetic diversity in wild barley was revealed by both nuclear- (2,505 SNP, 24 nSSR) and chloroplast-derived (5 cpSSR) markers in 256 widely-sampled geo-referenced accessions. Results were compared with MaxEnt-modelled geographic distributions under current, past (Last Glacial Maximum, LGM) and mid-term future (anthropogenic scenario A2, the 2080s) climates. Comparisons suggest large-scale post-LGM range expansion in Central Asia and relatively small, but statistically significant, reductions in range-wide genetic diversity under future climate. Our analyses support the utility of ecological niche modelling for locating genetic diversity hotspots and determine priority geographic areas for wild barley conservation under anthropogenic climate change. Similar research on other cereal crop progenitors could play an important role in tailoring conservation and crop improvement strategies to support future human food security.

## Introduction

Ecological niche modelling of the distributions of crop wild relatives in present, past and future climates can provide important insights into the past evolution and future trajectories of crop progenitors and domesticates [1]. Non-overlaps between current and predicted future distributions may reveal populations at particular threat from anthropogenic climate change [2]. At the same time, it has been suggested that overlaps between modelled past and present distributions may indicate refugial areas rich in genetic diversity, although this is a theory that requires wider validation [3]–[5]. In both instances, distributional differences may indicate wild genetic resources of particular importance for conservation and for breeding of the domesticated crop, in order to respond to new environmental pressures [6].

Wild barley (*Hordeum vulgare* ssp. *spontaneum*), the progenitor of the agriculturally important domesticated *H. vulgare* ssp. *vulgare* [7], provides an excellent opportunity to explore the utility of ecological niche modelling for supporting conservation and use. One reason is that its extensive natural distribution, which covers a range of environments across the Fertile Crescent and Central Asia [8], has been widely sampled for seed. This seed has been made available for genotyping and is an important resource for characterising genetic variation, to assist the cultivated crop to respond to anthropogenic climate change and other production challenges [9], [10]. Another reason for wild barley's utility is that a wide range of molecular tools are available to describe genetic diversity. These tools include single nucleotide polymorphisms (SNPs [11]), nuclear simple sequence repeats (nSSRs [12]) and chloroplast simple sequence repeats (cpSSRs [13]) that were developed initially for studying cultivated barley, but can also be used to characterise the wild resource. If centres of diversity in wild barley (as described by these tools) are spatially coincident with habitat common to past and present modelled distributions, then this would support the utility of niche modelling for locating genetic refugia. On the other hand, if centres of maximum variation are outside areas of common past-present habitat, then the utility of niche modelling for locating genetic diversity would be weakened.

In this paper, we explore this issue by combining ecological niche modelling, based on the MaxEnt procedure [14], with a spatial analysis of SNP, nSSR and cpSSR data sets, using various geographic information systems [15], [16], [17]. Our intention is to build a greater understanding of the impacts of climate change on wild barley, and to provide information to help manage natural stands better in the context of environmental change. In turn, this will support breeding to adapt the domesticated barley crop to future climate. Our analysis is based on a range-wide, fully geo-referenced collection of 256 wild barley accessions sampled from 19 countries, and involves distribution modelling under three conditions: current climate, climate at the Last Glacial Maximum (the LGM) and future climate for the 2080s under anthropogenic scenario A2. We begin to explore the possible utility of linkage disequilibrium analysis for discriminating between alternative hypotheses for describing and explaining spatial genetic structure, and discuss the merits and limitations of the methods we employ. An approach similar to that described in this paper could be used to improve the management of other progenitors of domesticated cereals originating in the Fertile Crescent and Central Asia. This is important for supporting future global human food security in the context of anthropogenic climate change [18].

## Materials and Methods

### The Wild Barley Collection

**Figure 1:**
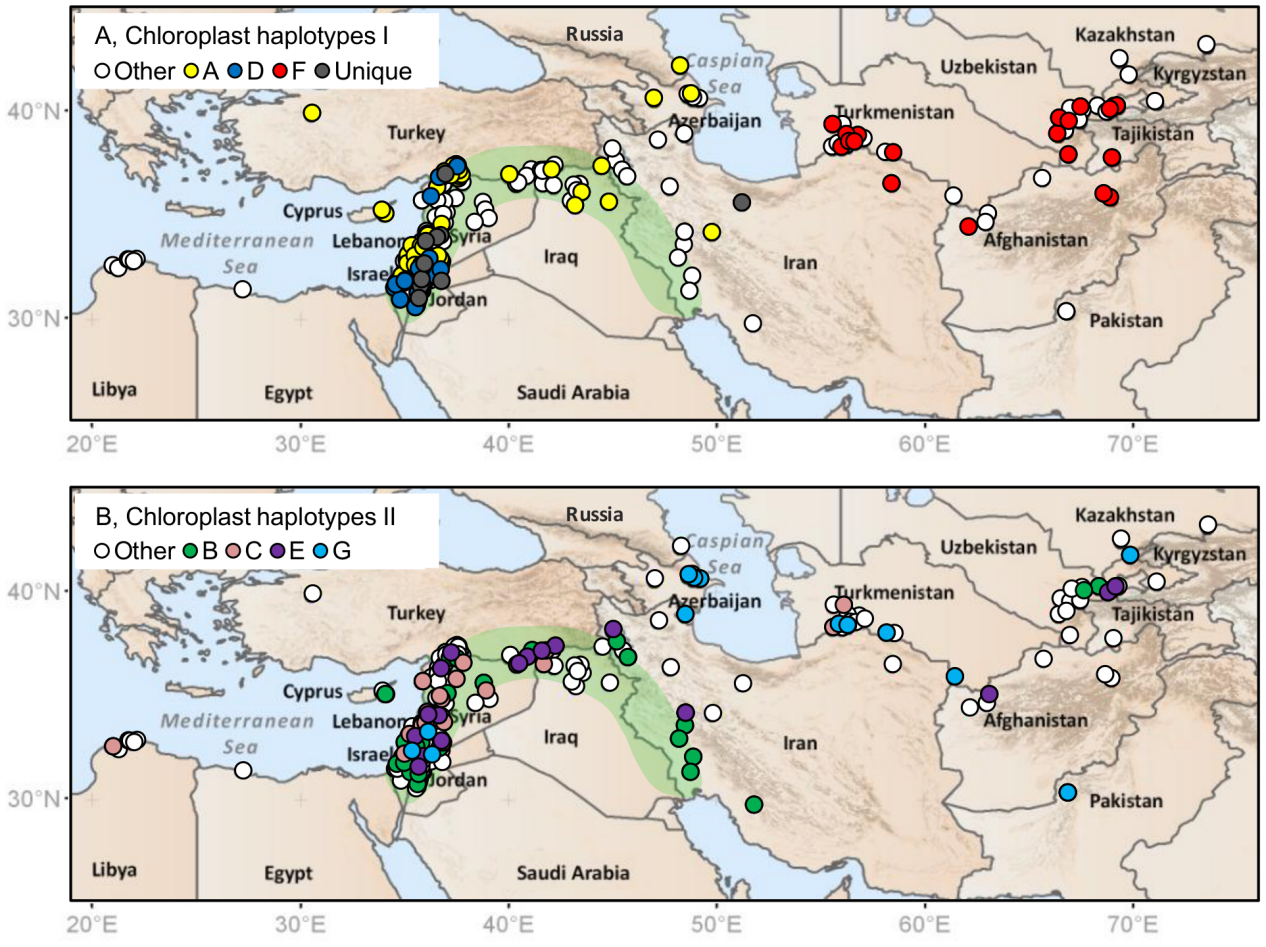
256 wild barley accessions sampled for genetic analysis and ecological niche modelling. Sampling covered 19 countries and much of the geographic range of wild barley. Superimposed on the positions of accessions are cpSSR haplotype designations for seven common haplotypes (frequency ≥0.05 across all accessions). A, distribution of three common, clearly geographically-differentiated, haplotypes. The distribution of 10 unique haplotypes is also shown. B, distribution of the other four common haplotypes. In both A and B, other sampled accessions are indicated by white circles (‘Other’). In total, 31 chloroplast haplotypes were revealed, as described in the Results and Discussion. The approximate dimensions of the Fertile Crescent, a region considered crucial in the development of agriculture and where dense stands of wild barley can occur [8], are indicated by green shading for reference purposes (see [26] for a discussion of barley domestication). The coordinates of sampled accessions and full chloroplast haplotype data are given in Table S1.

The Wild Barley Diversity Collection (WBDC) is the most comprehensive geo-referenced collection of *H. vulgare* ssp. *spontaneum* currently subject to wide characterisation [19]. Our sampling of 256 individuals from the WBDC included 19 countries and was designed to cover as much of the accepted natural distribution of the taxon as possible [8]. Sampling extended from North Africa through the Fertile Crescent into Central Asia (Fig. 1 and Table S1). Sampling did not include Tibet, where wild barley very different from that to the west is found [20], [21], as very few geo-referenced samples (as required for ecological niche modelling purposes) are available from there. Most accessions originated from the International Center for Agricultural Research in the Dry Areas (ICARDA), Aleppo, Syria, and were assembled by the former gene bank curator there, Dr Jan Valkoun. The majority of accessions were sampled from the wild in the fifty-year period 1953 to 2002, especially in the ten-year periods of 1983 to 1992 and 1993 to 2002 (79 and 101 accessions, respectively). These collection periods correspond well with when the weather station data that are used to support the interpolation of bioclimatic variables for ecological niche modelling were obtained (see below, [22]). For some accessions with early collection dates, latitudes and longitudes used in the current study are based on the interpretation of passport site-description data rather than actual given GPS coordinates. These accessions are therefore likely to be less precisely located.

### Assembling Molecular Marker Data Sets

Three molecular marker data sets were analysed in the current study. First, SNP data derived from two Illumina barley oligonucleotide pool assay platforms were used (see [11], [23] for a description of these platforms, referred to as BOPAs 1 and 2 or collectively as BOPA SNPs). Here, a subset of 2,505 mostly chromosome-position-mapped BOPA SNPs from an existing study on the WBDC ([24], to investigate disease resistance traits) was used. ‘Ascertainment bias’ can confound the interpretation of BOPA SNP results when comparing domesticated and wild barley genetic resources [25]. In the current study, however, which only involved comparing different portions of wild barley's range, no significant confounding effect is expected (see discussion in the study by Russell et al. [26], which compared landrace and wild barleys in the Fertile Crescent using BOPA SNPs). Second, we characterised variation *de novo* at 24 of the barley nSSR loci described by Ramsay et al. [12], using the methods given there. Third, we determined variation *de novo* at five of the cpSSR loci designed for *Hordeum* by Provan et al. [13], using the methods of Comadran et al. [27]. A list of all 2,534 loci used in the current study is given in Table S1.

### Analysing Molecular Marker Data

#### Spatial autocorrelation analysis

Spatial autocorrelation analysis using SPAGeDi [28] was undertaken to assess the relationship between inter-individual genetic identities of the 256 tested wild barley accessions and geographic distances. Separate analyses were carried out for BOPA SNPs, nSSRs and cpSSRs. Ritland's [29] kinship coefficient was employed to quantify average pairwise genetic identity based on 20 geographic distance classes of equal sample size. Whether or not individual kinship values were different from expectations (under a random spatial distribution of genetic variation) was assessed by a randomisation test with 1,000 permutations. Kinship values were regressed against the natural logarithm of distance classes to estimate the overall extent of spatial genetic structure. The significance of the regression slope was determined by 1,000 random permutations of locations.

#### STRUCTURE analysis

STRUCTURE analysis was not designed for predominantly inbreeding species such as barley, but it has been widely applied to cultivated and wild barley populations to reveal interesting genetic features (see discussion in [26]). Here, BOPA SNP and nSSR data sets were each analysed with STRUCTURE 2.2 [30] to assign accessions to one of *K* groups for different values of *K*. Each analysis was based on 25,000 ‘burn-in’ replications and a further 25,000 Markov chain Monte Carlo steps (initial trial runs indicated that these numbers of replications were sufficient to ensure the convergence of key parameters). After trial runs, *K* was set at five because log Pr(*X*/*K*) values in STRUCTURE had started to plateau at this point [30]. (Note that for our purposes it is more important to capture the major genetic divisions within data sets than to determine an ‘absolute’ value for *K*.) The ‘no admixture’ model in STRUCTURE was used to assign single states to individuals. Other analysis options were kept at default settings. STRUCTURE was run five times for each data set and the most common group assignments used as the basis for the interpretation of results (most accessions placed in the same groups in separate runs).

#### Circular neighbourhood analysis

To overlay genetic diversity onto geographic maps we employed DIVA-GIS 7.3 [15] (www.diva-gis.org) and ArcGIS 10 [16] (www.esri.com/software/arcgis/). Two approaches were used, the first based on allelic (or haplotype) richness and the second based on *K* groupings. In the first, allelic (BOPA SNP and nSSR) and haplotype (cpSSR) richness estimates were calculated for groups of accessions. Groups were circumscribed using a circular neighbourhood diameter of four degrees and a grid size of 30 minutes (method described in [6]). This allowed us to capture sufficient collection sites within neighbourhoods to estimate genetic parameters with some confidence. To account for varying sampling intensity in geographic space, which otherwise affects diversity estimates [31], rarefaction to a sample size of 10 individuals in neighbourhoods was undertaken using ADZE [17]. In the second approach, *K* groupings (*K* = 5) revealed by STRUCTURE for BOPA SNPs and nSSRs were used instead of allelic/haplotype richness estimates, in order to reveal genetic differentiation at a local geographic scale. Apart from this, the method of analysis was the same as applied in the first approach.

Circular neighbourhood analysis with rarefaction, as conducted in both the above approaches, has the advantage of allowing unbiased comparisons of genetic diversity across geographic space. However, it necessarily excludes accessions from analysis where sampling intensity is low. In the current study, a total of 38 accessions were thereby excluded (including from North Africa, southwestern Iran, Afghanistan and Azerbaijan).

### Ecological Niche Modelling

Although MaxEnt [14] has a number of well-documented limitations [32], it is reported to predict the natural distributions of plants well when based on ‘presence only’ location data compared to other ecological niche modelling approaches [33]–[35]. This is especially so when modelling is based on location data from a limited number of sites (less than 700 [34]). We therefore employed MaxEnt 3.3.1 to model geographic distributions for wild barley under current, past and future climates, in a manner similar to van Zonneveld et al. [36]. This involved extracting data for 19 bioclimatic variables for each of the 256 wild barley accession collection sites from WorldClim [22] (www.worldclim.org/) (extracted values for variables listed in Table S1). These 19 variables are derived from monthly temperature and rainfall values and include seasonality and limiting environmental factors. Our modelling was bounded by longitudes of 18.63 and 80.42 degrees east, latitudes of 21.58 and 47.96 degrees north. The output of MaxEnt is a grid map with each cell assigned a probability of taxon presence [37]. Modelled distribution areas were restricted to the threshold suitability value of maximum training sensitivity plus specificity recommended by Liu et al. [38]. In total, 13 accessions were excluded from modelling because they were identified to occur at ‘outlier’ sites (see Table S1).

For the LGM (∼21,000 years before present), modelling was based on CCSM and MIROC models (available at WorldClim, [2]; the results of models were averaged to provide overall estimates). The LGM is believed to have been an influential period in determining contemporary patterns of genetic variation in many plant species and much modelling of past distributions has therefore been based on it [2], [3], [5]. For future climate, modelling was based on the 2080s period (2070 to 2099) and the medium- to high-emission trajectory A2 for anthropogenic global warming. The 2080s A2 scenario has been widely used in modelling to provide insights on a timescale and threat level that is useful for planning purposes [39], [40]. Nineteen general circulation models (GCM) were used for future climate (again, results were averaged across models). Data on future climate projections were provided by the CGIAR Climate Change, Agriculture and Food Security Research Programme (CCAFS) and downscaled with the Delta method [39].

For current, past and future distribution modelling, 2.5-minute downscaled climate layers were employed, which is the same resolution as used by Waltari et al. [2] for past-climate distribution projections. Based on the overall geographic scale of our sample range, we consider this degree of resolution sufficient for our study.

## Results and Discussion

### Isolation-by-Distance Alone Does Not Explain the Observed Genetic Structure in Wild Barley

Individual scores for all 256 wild barley accessions for 2,505 BOPA SNPs, 24 nSSRs and five cpSSRs are provided in Table S1. The overall quality of the data was high, with the mean level of missing data (including ambiguous calls) across all markers less than 1%. 2,363 BOPA SNPs were polymorphic and all 24 nSSRs and 5 cpSSRs. A mean of 16.7 alleles per locus (ranging from 3 to 54) was revealed at nSSRs. Length variation at cpSSR products indicated a series of single base differences, with combined data (summing differences across separate products) revealing 31 haplotypes. Seven haplotypes could be defined as common (A to G, occurring at a frequency ≥0.05), while 10 were unique. Compared to haplotype A, haplotypes B, C, D and E differed by a single nucleotide length at one cpSSR product, while haplotypes F and G differed by single nucleotide length differences at two products. Of the common haplotypes, A, D and F showed very clear geographic structuring but the others did not (compare Figs. 1A and B). Haplotype A occurred throughout the Fertile Crescent but not further east, D occurred only in the Eastern Mediterranean region of the Fertile Crescent, while F occurred only in Turkmenistan and further east.

**Figure 2:**
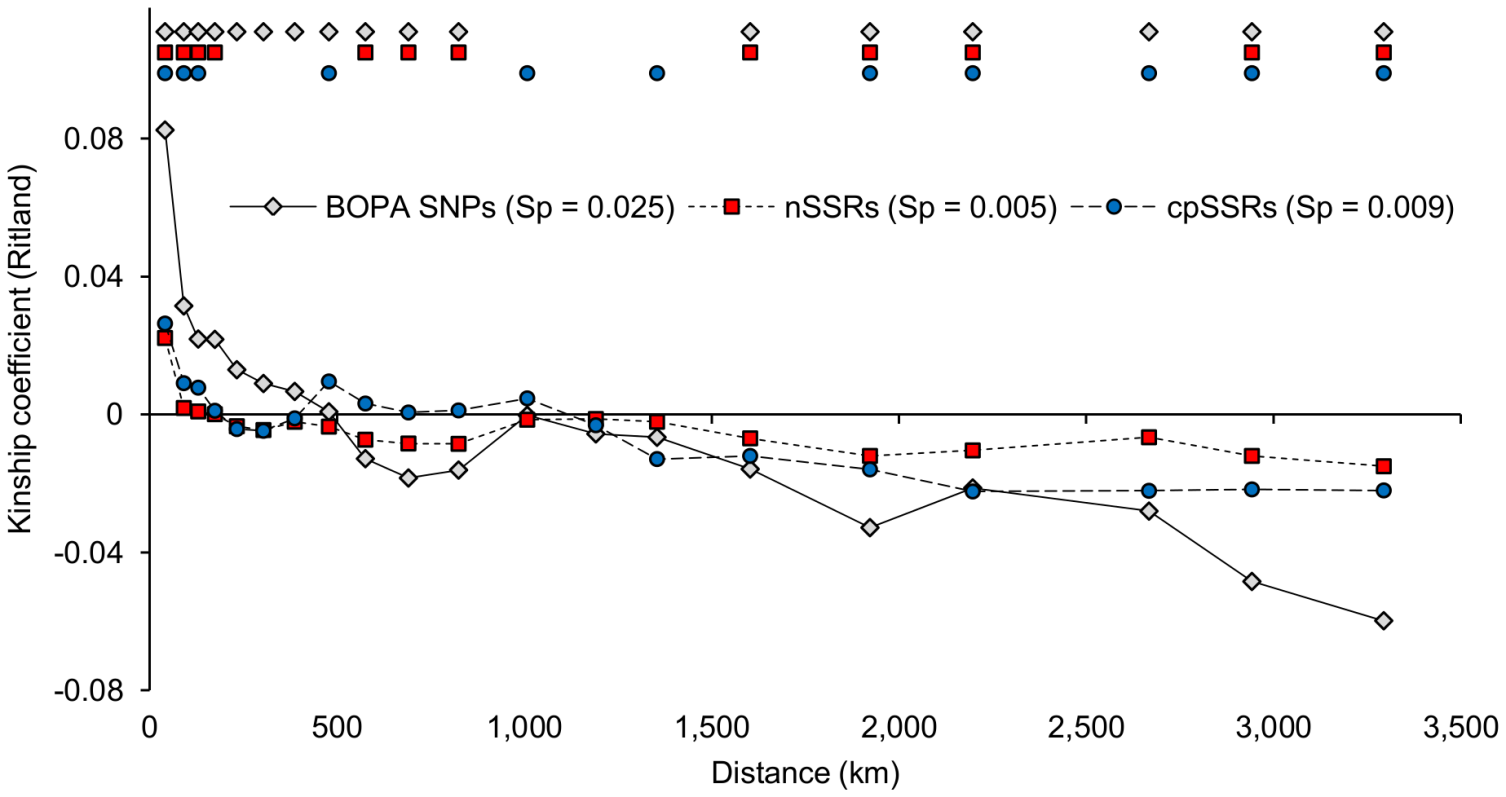
Spatial autocorrelation analysis profiles for wild barley accessions based on BOPA SNPs, nSSRs and cpSSRs. Geographic distances on the *x*-axis are the mean values of distance classes. The symbols at the top of the figure mark observations significantly larger or smaller (*P*≤0.01) than the average for distance classes. Values for the *Sp* statistic, calculated from the regression slope of the graph and the kinship coefficient of the first distance class [76], are also shown. Placing all three data sets on the same graph allows profiles to be compared. Increases in similarity at a distance class of around 1,000 km, and an earlier additional increase for cpSSRs at around 500 km, illustrate that a simple isolation-by-distance model is not sufficient to describe genetic variation in wild barley.

**Figure 3:**
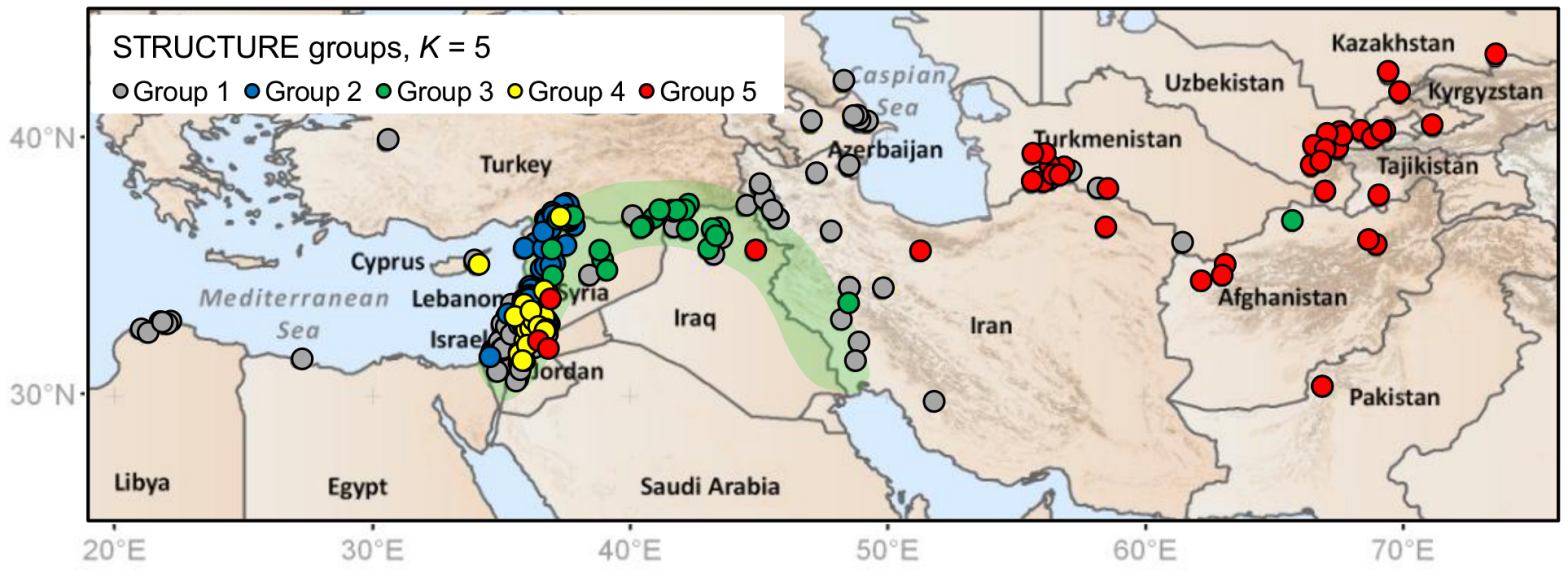
STRUCTURE group assignments for individual wild barley accessions. The results shown are based on *K* = 5 and for BOPA SNPs. The results for nSSRs (not shown) were similar. Results correspond with spatial autocorrelation analysis (Fig. 2) in describing a more complex genetic structure in wild barley than might be expected with a simple isolation-by-distance model.

Spatial autocorrelation analysis has been widely applied to assess genetic structure in plant species and to describe deviations from isolation-by-distance expectations (e.g. [78], [79]). It can also be a useful method for comparing different molecular marker data sets compiled on the same taxon, as we do here for wild barley (Fig. 2). In our analysis, a degree of geographic-based genetic structure is evident for BOPA SNPs, nSSRs and cpSSR haplotypes (*P*<0.01 in a test for overall structure in each case). The decrease in similarity observed with geographic distance is, however, not a simple trend for any of our three data sets. Differences in profiles are also observed between marker types. For both BOPA SNPs and nSSRs, an increase in similarity at a distance class of around 1,000 km is observed, after which similarity declines again. For cpSSR haplotypes, an obvious increase in similarity is also observed at a distance class of around 500 km. Spatial autocorrelation analysis therefore indicates that a simple isolation-by-distance model does not fully explain genetic structure across the geographic range of wild barley tested (as indicated also, e.g., by the distribution of accessions among BOPA SNP STRUCTURE groups, as shown in Fig. 3). In such situations, climate change-related expansions and contractions in range could have a role in determining patterns of variation [3], [5].

The differences we observed for spatial autocorrelation analysis profiles for nuclear BOPA SNPs and nSSRs compared to maternally-inherited cpSSRs may indicate the more restricted role of seed when compared to pollen in gene flow (even though wild barley is predominantly self-pollinated and so pollen-mediated gene flow is expected to be relatively low [41]). The smaller effective population size of the organellar genome compared to the nuclear genome may also be a factor in determining the differences observed [42].

### The Spatial Distribution of Genetic Variation Corresponds With Niche Modelling in Locating Diversity Hotspots and is Consistent With Post-LGM Expansion in Central Asia

**Figure 4:**
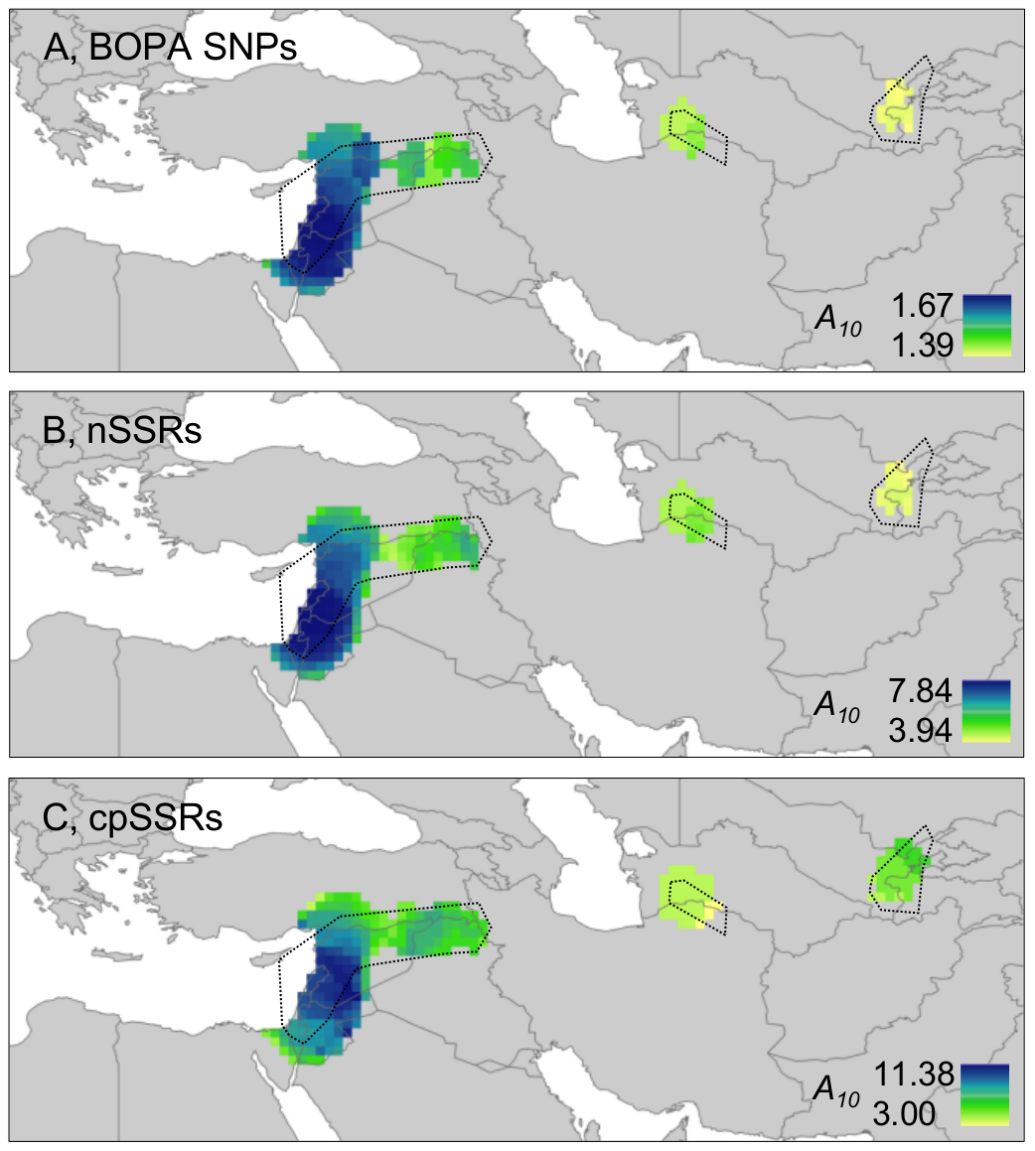
Allelic (A and B) and haplotype (C) richness (*A_10_*) maps for wild barley. BOPA SNPs, nSSRs and cpSSRs all indicate the Eastern Mediterranean region as more diverse (highly diverse areas = dark blue) than Central Asia. As expected, nSSRs with high allelic diversity and cpSSRs with multiple haplotypes reveal relatively higher richness values within neighbourhoods (*A_10_* as high as 7.84 and 11.38, respectively) than biallelic BOPA SNPs (maximum *A_10_* = 1.67). Not all of the original sample range could be included in analysis because of the required minimum sampling intensity to calculate a standardised diversity value (see Materials and Methods; compare the current figure with Fig. 1). Accessions included in analysis in a particular geographic area are circumscribed by a dotted line. A, 2,426 from 2,505 BOPA SNPs used in calculations (SNPs excluded with ≥25% missing data in one or more grid cells); B, all 24 nSSRs used in calculations; C, all cpSSR haplotypes used in calculations.

**Figure 5:**
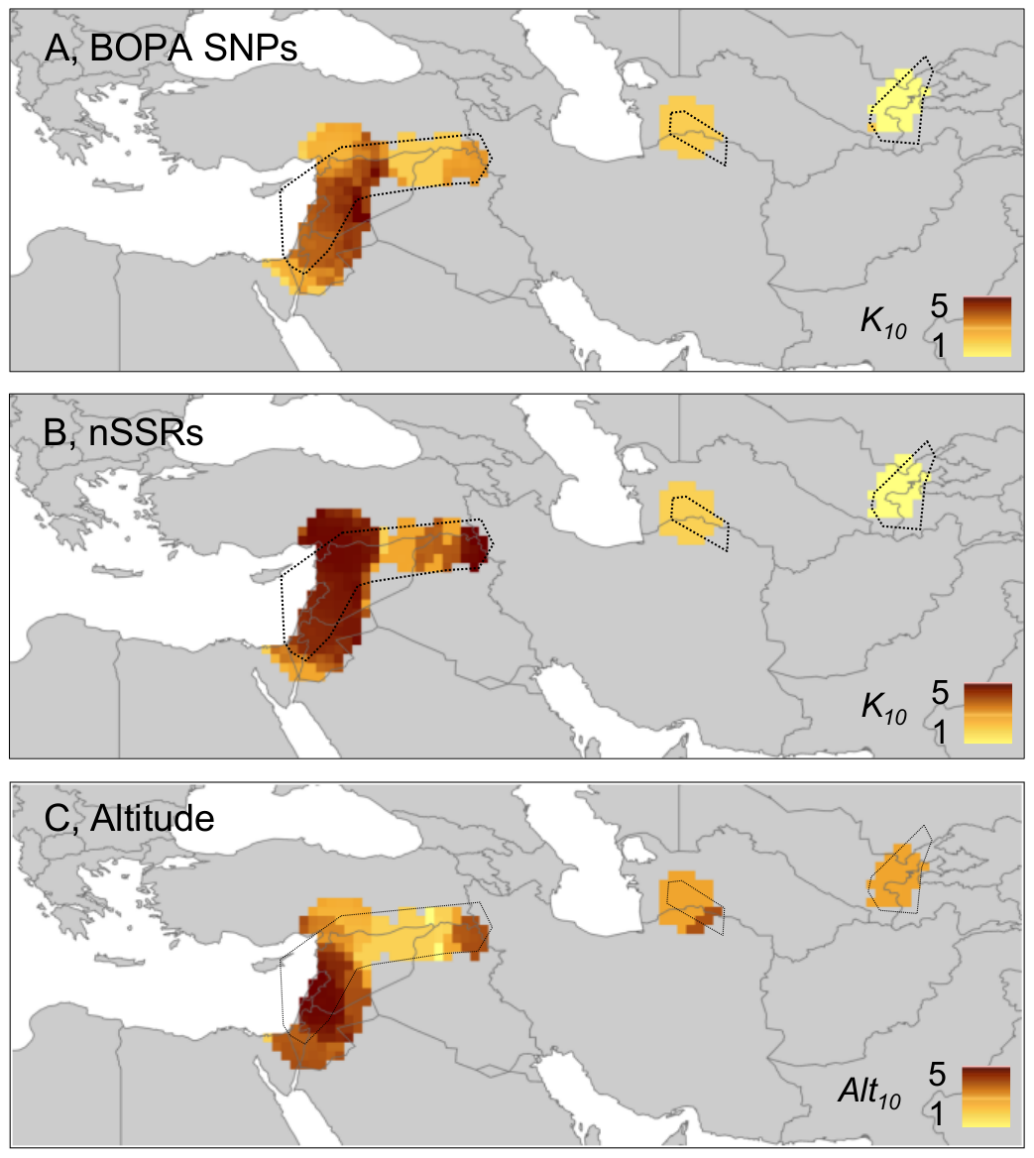
STRUCTURE group richness (A and B, *K_10_*) and ‘altitude richness’ (C, *Alt_10_*) maps for wild barley. A and B, richness estimates for BOPA SNPs and nSSRs, respectively, *K* = 5 in STRUCTURE analysis. Both marker sets indicate the Eastern Mediterranean region as more diverse (highly diverse areas = dark brown) than Central Asia. C, ‘altitude richness’ of wild barley sample sites, based on five altitude categories (<200 m, 200 to 600 m, 600 to 1,000 m, 1,000 to 1,400 m, >1,400 m). Altitude data provide an indication of environmental heterogeneity and were downloaded from WorldClim (www.worldclim.org/; values given in Table S1). Unlike the 19 bioclimatic variables used elsewhere in the current study, altitude data are actual values rather than interpolations from weather station records, so they are particularly appropriate for assessing real environmental heterogeneity [22], [77]. Altitude richness estimates indicate sample points in the Eastern Mediterranean region as more diverse than those in Central Asia. Not all of the original sample range could be included in analyses because of the required minimum sampling intensity to calculate standardised diversity values (see Materials and Methods; compare the current figure with Figs. 1 and 3 [individual STRUCTURE *K* group assignments], see also Fig. 4). Accessions included in analyses in a particular geographic area are circumscribed by a dotted line. The analysis to generate ‘altitude richness’ was carried out in the same way as for STRUCTURE group richness, except ‘altitude category’ substituted for ‘STRUCTURE group’.

Geographic information systems are underutilised in genetic diversity studies, but they can be very effective in expressing variation in geographic and environmental space [43]–[45]. Our analysis is the first on wild barley to use circular neighbourhoods with rarefaction to account for differences in sampling intensity across geographic space. These differences otherwise skew the visualisation and interpretation of genetic diversity, as illustrated by nSSR analyses of cacao (*Theobroma cacao* [5]) and the cherimoya fruit tree (*Annona cherimola* [6]) in South America. The results of our analyses of wild barley are given in Figures 4 and 5. Figure 4, based on allelic/haplotype richness estimates, demonstrates that BOPA SNPs, nSSRs and cpSSRs all provide similar profiles of diversity across geographic space. In each case, higher sample-size-corrected values of richness were observed in the Eastern Mediterranean region than in Central Asia. Circular neighbourhood analysis based on chloroplast haplotypes therefore clearly corresponds with the distribution of unique haplotypes shown in Figure 1 (nine of 10 unique haplotypes occurred in the Eastern Mediterranean region). Figure 5 (A, B), which shows levels of BOPA SNP and nSSR diversity based on *K* group richness, also indicates higher diversity (greater genetic differentiation at a local level) in the Eastern Mediterranean than in Central Asia (as also evident from individual *K* group assignments in Fig. 3).

Our findings are consistent with the limited previous molecular marker research (uncorrected for sampling intensity) comparing wild barley from the Eastern Mediterranean region and environs with Central Asia. For example, Volis et al. [46] measured lower variation in wild barley in Central Asia (samples from Turkmenistan only) than in the Eastern Mediterranean using isozymes, while Fu and Horbach [47] found the same based on nSSRs. Our analysis provides comprehensive evidence to reinforce these observations and confirms the status of the Eastern Mediterranean wild barley stands as important resources for conservation and evaluation [9], [10].

**Figure 6:**
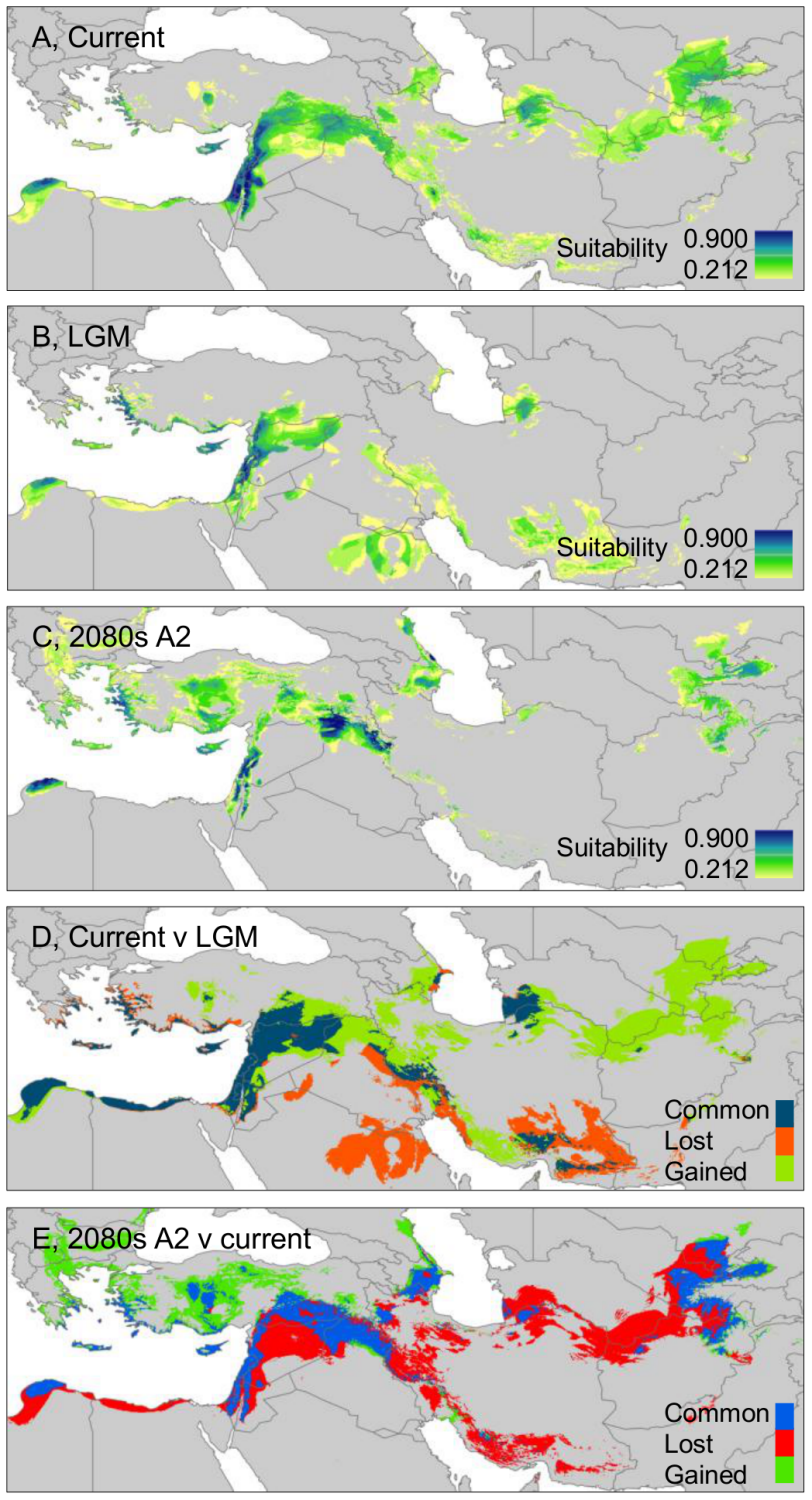
Potential wild barley distributions in current (A), past (B) and future (C) climates. Distributions are based on ecological niche modelling using MaxEnt (see Materials and Methods). D, differences between current and past modelled distributions, including areas lost and gained since the LGM. E, differences between future and current modelled distributions, including areas expected to be lost and gained by the 2080s. Note that past and future distribution maps take no account of rises or falls in sea levels or of other water bodies, and that these distributions are shown superimposed on current country boundaries.

Our intention in this study is to compare patterns of genetic variation in wild barley with the modelled distributions of the taxon under all three conditions of current, past and future climates, something which to our knowledge has not been undertaken before for any member of the genus *Hordeum*. The results of our ecological niche modelling are presented in Figure 6, from which several interesting observations can be drawn in relation to the genetic data, as set out in this section for the current-past climate comparison and in the next section for the current-future climate comparison.

Considering first the modelled present-day geographic distribution of wild barley, visual assessment confirms that the accessions included in our study for genetic analysis provide good coverage of most of the taxon's range in the Fertile Crescent and Central Asia (compare Fig. 6A with Fig. 1). The most obvious exception is the ‘peak’ of the Fertile Crescent in southern Turkey, where it is known that important stands of wild barley occur [8]. Although not sampled in this study, these Turkish stands should be incorporated in future work. A comparison of the modelled present-day geographic distribution of wild barley with that projected for the LGM (Figs. 6B, D) suggests that since the LGM suitable habitat has been lost in areas that include southeastern Iran and northern Saudi Arabia. At the same time, the comparison indicates that at the LGM wild barley was, just as it is now, widely present in the region bordering the Eastern Mediterranean coast. A comparison of current and past modelled distributions also indicates habitat gains since the LGM. Particular areas identified in this regard include the northern Iraq portion of the Fertile Crescent and, especially, a large part of Central Asia.

The apparent relatively recent range expansion of wild barley in Central Asia as revealed by ecological niche modelling is consistent with our findings of lower levels of genetic diversity in the region compared to the Eastern Mediterranean, in which latter region it can be postulated that a continuous presence of the taxon has led to the accumulation of genetic diversity (as shown in Figs. 4, 5A and B) there. The correspondence between past-present ecological niche modelling and our analysis of spatial genetic diversity in wild barley has important implications, as it supports the utility of niche modelling as a tool for identifying genetically diverse and potentially refugial areas. There are, however, other possible reasons why different levels of genetic diversity are observed across wild barley's range. The Eastern Mediterranean region (as represented, e.g., by altitudinal variation for the accessions included in the current study, see Fig. 5C) is, for example, particularly environmentally heterogeneous. This may have allowed more genetic variation to develop and accumulate there compared to Central Asia without recourse to an explanation based upon post-LGM macro-geographic range adjustment. We are not able to distinguish between these alternatives (or, indeed, to understand whether a combination of both range expansion and environmental heterogeneity are important) for determining the current pattern of spatial genetic diversity observed in wild barley. Nevertheless, one interesting feature of our data that deserves further exploration in this regard is the level of linkage disequilibrium (LD) between chromosome-position-mapped BOPA SNP markers in different parts of wild barley's range, as we relate below.

**Figure 7:**
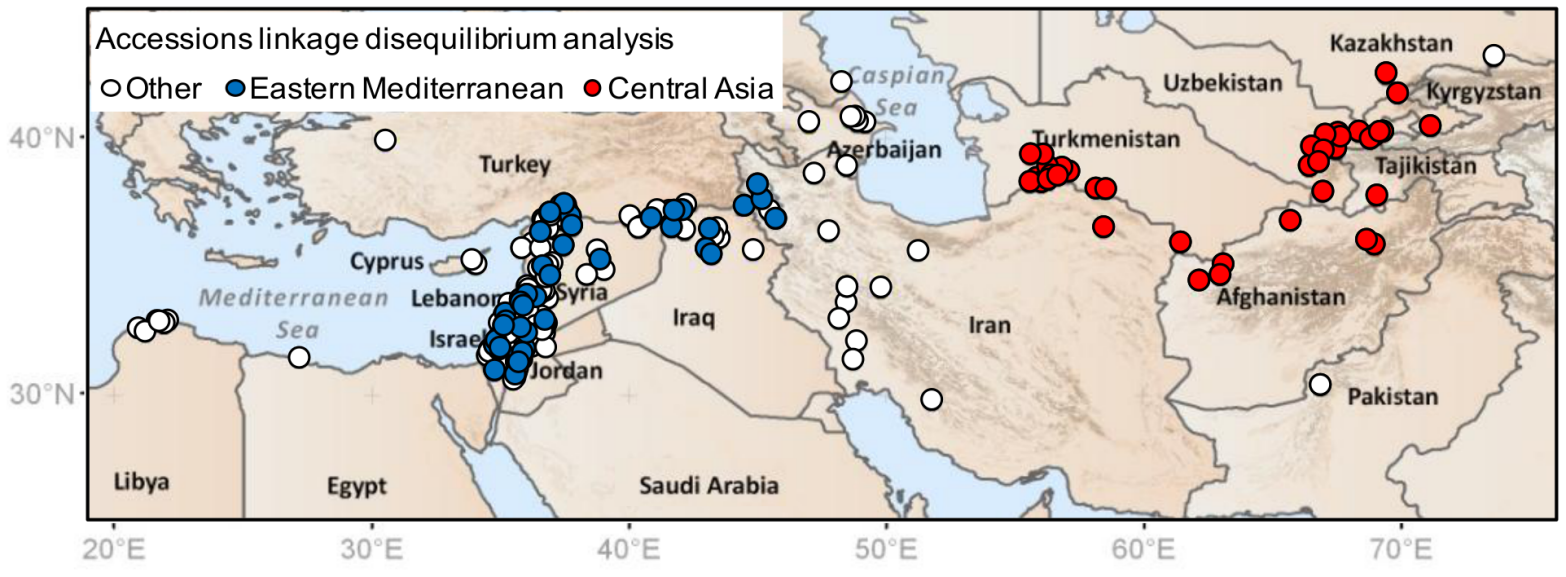
Locations of wild barley individuals sub-sampled from two regions for testing of linkage disequilibrium (LD). For LD assessment, forty accessions were chosen at random from the Eastern Mediterranean and Central Asia regions, across an approximately equal-dimensioned geographic area to minimise confounding sample size and dimensional effects in analysis (a significant issue in LD calculations [26]). In the case of the Eastern Mediterranean region, to ensure similar geographic coverage to Central Asia, sampling was extended eastward away from the coast below the peak of the Fertile Crescent into northwestern Iran. The coordinates of the accessions sampled for LD analysis are given in Table S1. Compared to the Eastern Mediterranean sub-sample, that from Central Asia had a less variable environment across accession collection sites (see Fig. 5C and the bioclimatic variables given in Table S1). Furthermore, climate modelling suggested a much greater proportion of accessions in the Central Asian sub-sample to be associated with range expansion since the LGM (see Fig. 6D and Table S1). Consistent with genetic diversity levels expressed on maps (Figs. 4, 5A, 5B), the latter sub-sample also had lower nuclear diversity according to FSTAT 2.9.4 [61] calculations (nSSR allelic richness for the Eastern Mediterranean sub-sample = 10.44, for Central Asia = 7.44, corrected by rarefaction to a sample size of 36 complete genotypes across all nSSRs; *P*<0.001 based on a two-tailed *t*-test of individual locus allelic richness values undertaken in EXCEL). Another factor that can confound LD comparisons is differences in allele frequency distributions between samples. We therefore tested allele frequency profiles for our two sub-samples, and found them to be similar (proportion of markers with a minimum minor allele frequency between 0.1 and 0.3 was 0.493 and 0.499 for the Eastern Mediterranean and Central Asia areas, respectively).

**Figure 8:**
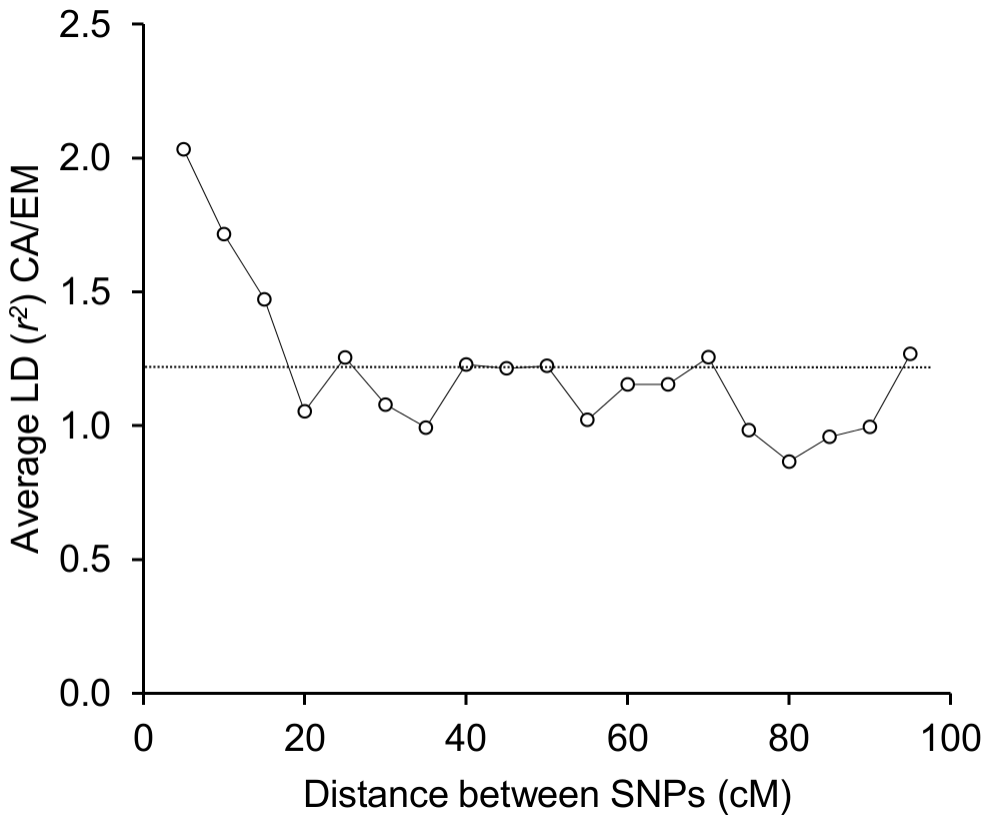
Comparison of mean linkage disequilibrium (LD) (*r^2^*) values across all wild barley chromosomes. CA = Central Asia, EM = Eastern Mediterranean sub-samples. Comparisons are for BOPA SNPs at five cM intervals (SNPs 0 to 5 cM apart, 5 to 10 cM apart, etc.). The dotted line indicates the average value of all plotted comparisons. The graph indicates that when compared to longer pairwise SNP distances, LD estimates for shorter pairwise SNP distances are relatively higher in the Central Asia sub-sample than in the Eastern Mediterranean sub-sample. The difference between sub-samples appears to be lost after about 15 cM.

To explore LD, we undertook a further analysis based on two sub-samples of our wild barley accessions taken to represent the Eastern Mediterranean and Central Asia regions, as shown in Figure 7. These sub-samples, each of 40 individuals, represent approximately balanced sets of material (as explained in the legend to Fig. 7) for LD comparison. Compared to the Eastern Mediterranean sub-sample, the Central Asian sub-sample is of lower genetic diversity, comes from a more uniform environment and has a much higher proportion of accessions collected from habitat established (apparently) since the LGM. For these sub-samples of accessions, we then calculated LD for pairs of chromosome-mapped BOPA SNPs falling into different centimorgan (cM) distance categories along each of barley's seven chromosomes. Estimates for LD were based on 487 SNPs (the number of mapped SNPs on each chromosome ranged from 57 to 80) with a minimum minor allele frequency of 0.1 in both sub-samples, while the chromosome distance interval for making pairwise comparisons was set at five cM (so comparisons for paired SNPs 0 to 5 cM apart, 5 to 10 cM apart, etc.). The level of LD was estimated with *r^2^* values (the squared correlation of allele frequencies [48], [49]) using DNASP 5.00.07 [50] with all SNPs assigned homozygous status (i.e., no intra-locus component in analysis). Finally, once *r^2^* values were generated, they were compiled into mean values for chromosome distance categories (a minimum of 10 observations for a distance interval were required before assigning a mean value) for each chromosome, and then averaged across chromosomes, in EXCEL. Results were then expressed in graphical form comparing LD estimates across sub-samples (mean *r^2^* Central Asia sub-sample/mean *r^2^* Eastern Mediterranean sub-sample) (Fig. 8).

Our comparison of LD estimates indicates that at shorter chromosome distance intervals values of LD are relatively higher in Central Asia than in the Eastern Mediterranean, but that with increased distance along chromosomes values become more equal. Linkage disequilibrium values are difficult to interpret because of the many influencing factors, including population structure, selection pressures, mating patterns and changes in population size and range [26], [51]–[53]. In the absence of confounding factors, however, our observations are consistent with a relatively recent geographic expansion in Central Asia that has not allowed for enough time for recombination between proximate paired markers to result in equilibrium (convergence) between them [54]–[56]. Although interesting, we stress that this interpretation of the results of our LD analysis is speculative and must be treated with caution. For example, the selfing rate for wild barley can vary across populations (see [41] and [57] for Israeli and Jordanian populations, respectively), and if wild barley stands in Central Asia were more highly selfed [58], this could also explain why LD for proximate markers was higher there than in the Eastern Mediterranean. Clearly, more research on this topic is required.

### Niche Modelling Suggests Small, But Statistically Significant, Losses of Genetic Diversity in Wild Barley Under Mid-Term Anthropogenic Climate Change

A comparison of present-day plant distributions with predictions for the 2080s is useful for devising responses to anthropogenic climate change [1], [32]. For example, areas of predicted habitat loss may be targets for the collection of seed that can then be stored in gene banks. In addition, locations where habitat is likely to be retained may be priorities for *in situ* conservation measures [59], [60]. Of most interest may be locations where habitat is predicted to be retained in geographic regions of general habitat loss. Our ecological niche modelling comparing the current distribution of wild barley with that predicted for the 2080s under the A2 emission scenario is shown in Figure 6 (C, E). The comparison suggests that suitable habitat will be lost in particular in large areas of Iran, northern Syria and in the border region of Afghanistan and Turkmenistan. At the same time, potential habitat will be gained most notably in parts of Turkey.

In order to calculate the possible losses in range-wide genetic diversity in wild barley associated with future habitat loss, we compared allelic richness at nSSRs for accessions predicted to be in shared future and current habitat (*N* = 155, i.e., excluding accessions in ‘lost’ habitat, as indicated in Table S1) with accessions in the current distribution (*N* = 243). We chose nSSRs as the estimator for this analysis because of their high allelic variability and hence sensitivity in describing diversity differences. Our analysis indicated a relatively small reduction in allelic richness under climate change (shared habitat, *A* = 14.88; current habitat, *A* = 15.88; estimates calculated in FSTAT 2.9.4 [61] and corrected by rarefaction to a sample size of 135 complete genotypes across all nSSRs). Although small, the difference was statistically significant (*P* = 0.012 based on a two-tailed *t*-test of individual locus allelic richness values undertaken in EXCEL). Further assessment, based on BOPA SNPs and cpSSRs, revealed a relatively modest 108 SNP alleles (4.6% of SNPs in the comparison) and four chloroplast haplotypes unique to 2080s ‘lost’ habitat. Our analysis therefore suggests that, overall, mid-term future losses in genetic diversity due to climate change are expected to be relatively low. A comparison of modelled current, past and future distributions (Fig. 6) suggests that in part this is because much predicted future habitat loss is in areas of putative post-LGM range expansion, where contemporary genetic variation is relatively low (e.g., in the border region of Afghanistan and Turkmenistan). On the other hand, shared future-current habitat includes much of the more genetically diverse putative LGM refugial regions. Of concern, though, could be future habitat loss in parts of northern Syria, where habitat is in common in the past-current comparison, but not in the future-current comparison (compare Figs. 6D, E).

It is important to consider a number of provisos when interpreting our findings. First, we have not considered in our analysis that existing wild barley populations in habitat that will be lost under climate change could migrate to (newly) environmentally-matched sites. Such migration will presumably be easier in areas with greater micro-geographic environmental heterogeneity, as the distances to be moved are then smaller. This would suggest migration is more feasible in the Eastern Mediterranean region (see Fig. 5C). Whether migrations are possible also depends on the level of human activity in wild barley's habitat. Human disturbance that provides opportunities for establishment could be beneficial, while modern agricultural practices that intensify crop production (excluding other plants from fields) and fragment wild habitat could be detrimental [62]. Second, in common with most other studies that compare present-day and potential future distributions to make conservation predictions (e.g., [5], [63]), our analysis does not consider the possibilities for the local adaptation of plant populations to new climatic conditions. There is little relevant research on this topic for wild barley, which ideally requires multiple time-interval-based monitoring of wild stands [64]. Nevo et al. [65], however, did assess genetic variation and phenotypic traits in the same 10 natural stands of wild barley in Israel sampled first in 1980 and then again in 2008. The authors observed some change in the distribution of nSSR alleles and larger changes in flowering times (accessions sampled in 2008 flowered significantly earlier under greenhouse conditions). These changes could indicate responses to climate change, although other explanations are also possible. Interestingly, the changes in wild barley nSSR composition observed by Nevo et al. [65] were much smaller than those found in wild emmer wheat (*Triticum dicoccoides*) populations included in the same study and sampled at the same dates. This suggests different responses to climate change by different cereals in the Eastern Mediterranean region. Third and finally, our current assessment was based on genetic markers that are presumably (mostly) neutral with regard to phenotype, so we are not able to determine whether or not there will be important losses in functional genetic diversity under anthropogenic climate change.

## Final Considerations

Our analyses are consistent with the view that climate change has played a role in determining the levels of present-day genetic variation observed in wild barley in different portions of its natural range. Our data support the utility of ecological niche modelling of current and past plant distributions for predicting geographic areas of high genetic diversity, and suggest limited future losses of genetic diversity in wild barley under mid-term future climate change. We have explored the use of chromosome-position-mapped SNPs for discriminating between different hypotheses to explain diversity patterns in wild barley, but more research is required on this topic, ideally using SNPs that have been physically positioned in the genome. With the promise soon of a complete genome sequence for barley (building on the current sequence assembly [66], [67]), the physical distances between the SNPs used in the current study will soon be available. This will allow more formal LD analysis of possible range expansions and contractions in relation to climate change. Further research on wild barley should also explore the ensemble forecasting of distributions based on both the differences between GCM and the multiple statistical methods available for species modelling [68], [69]. Modelling should also investigate the possible further downscaling of environmental data in predictions [70], which could provide greater accuracy [34], [71].

The wider utility of past-present ecological niche modelling for locating centres of genetic diversity in the Fertile Crescent and Central Asia regions could be tested by examining the wild progenitors of other important cereals located there [8]. Molecular marker data sets are available for comparison purposes (e.g., for einkorn wheat [*Triticum monococcum*] and emmer wheat [72], [73]), although more systematic assessments of genetic diversity are required based on fully geo-referenced samples. It would be interesting to model the distributions of different wild cereals at the time it is proposed that humans began to manage them significantly. How modelled distributions correspond with putative sites of first cultivation and first domestication [74] could then be explored. For example, modelling to understand distributions over the transition to the Younger Dryas (∼12,000 years ago) would be useful. This was a relatively cold and unfavourable period for humans in the Fertile Crescent region that is believed to be associated with early cultivation events, leading eventually to domestications [75]. Distribution modelling of wild barley combined with geographically coincident sampling and genetic analysis of wild and landrace accessions of the taxon (and of other cereals) throughout the region will provide important insights into domestication processes [26].

## Supporting Information

Table S1 **Geographic coordinates, environmental and genetic data for 256 wild barley accessions.**
(XLSX)
